# Midlife physical activity and amnestic MCI: Impact on hippocampal volume

**DOI:** 10.1002/alz.093331

**Published:** 2025-01-09

**Authors:** Lynette J Tippett, Jenna Merrick, Reece P Roberts, Catherine A Morgan, Tracy R Melzer, Ian J Kirk, Erin E Cawston, Joanna M Williams, Sarah M Buchanan, Nicholas J Cutfield, John C Dalrymple‐Alford, Tim J Anderson, The New Zealand‐Dementia Prevention Research Clinics, New Zealand

**Affiliations:** ^1^ School of Psychology, The University of Auckland, Auckland New Zealand; ^2^ Centre for Brain Research, The University of Auckland, Auckland New Zealand; ^3^ NZ Brain Research Institute, Christchurch New Zealand; ^4^ Department of Medicine, University of Otago, Christchurch New Zealand; ^5^ Department of Pharmacology, University of Auckland, Auckland New Zealand; ^6^ Department of Anatomy, University of Otago, Dunedin New Zealand; ^7^ Brain Health Research Centre, University of Otago, Dunedin New Zealand; ^8^ Department of Neurology, Southern District Health Board, Dunedin New Zealand; ^9^ Department of Medicine, University of Otago, Dunedin New Zealand; ^10^ School of Psychology, Speech and Hearing, University of Canterbury, Christchurch New Zealand

## Abstract

**Background:**

The projected doubling of dementia prevalence by 2050 highlights the critical importance of altering the dementia trajectory and associated burden. Modifiable risk factors account for 40% of dementia risk (Livingston et al, 2020), including physical inactivity in late life. Protective effects of physical activity (PA) may be greater during earlier stages of neuropathological change, either directly by influencing the brain, or, indirectly by protecting against hypertension, obesity, and diabetes. We investigated whether midlife PA (i) differs in participants spanning the continuum from healthy older adults to clinically diagnosed early AD, and (ii) is related to hippocampal volume later in life.

**Method:**

214 individuals from the New Zealand‐Dementia Prevention Research Clinics participated (31 healthy controls; 56 subjective cognitive decline; 107 amnestic mild cognitive impairment (aMCI) and 20 early AD dementia). Midlife physical activity was based on the Lifetime of Experiences Questionnaire, used to derive cumulative PA frequency scores, and weighted PA frequency‐by‐intensity composite scores. Hippocampal volume was computed from 3T MRI T1‐weighted images using SPM CAT12. ANCOVAs assessed between‐group differences on transformed PA variables. Groups were reduced to cognitively unimpaired (n = 87) and aMCI (n = 107) for multiple linear regressions that assessed whether midlife PA composite and frequency scores were associated with total hippocampal volume and whether these were moderated by gender and APOE status. Age, total intracranial volume and group were entered as predictors.

**Result:**

**T**here were no significant between‐group differences in transformed frequency or composite midlife PA measures (*p*‐values > .35). Midlife composite PA and frequency PA scores were associated with hippocampal volume, with associations moderated by gender (Beta gender X PA *p*‐values = .02 and .03), but not APOE status. Females showed the predicted positive association between midlife exercise and hippocampal volume, but males showed negative associations. Multiple regressions with only amnestic MCI participants revealed the same patterns of associations.

**Conclusion:**

These findings suggest that the effects of midlife PA on hippocampal volume are detectable later in life in aMCI. Negative associations for males may relate to previous findings (human and animal) suggesting that leisure‐time PA is protective for the brain, but not occupational or “involuntary” exercise.

